# Correlation between ELF–PEMF exposure and Human RPE Cell Proliferation, Apoptosis and Gene Expression

**DOI:** 10.18502/jovr.v16i2.9084

**Published:** 2021-04-29

**Authors:** Morteza Oladnabi, Mohammad Amir Mishan, Mozhgan Rezaeikanavi, Mehryar Zargari, Rouhallah Najjar Sadeghi, Abouzar Bagheri

**Affiliations:** ^1^Stem Cell Research Center, Golestan University of Medical Sciences, Gorgan, Iran; ^2^Ischemic Disorders Research Center, Golestan University of Medical Sciences, Gorgan, Iran; ^3^Ocular Tissue Engineering Research Center, Research Institute for Ophthalmology and Vision Science, Shahid Beheshti University of Medical Sciences, Tehran, Iran; ^4^Department of Clinical Biochemistry and Medical Genetics, Molecular and Cell Biology Research Center, Faculty of Medicine, Mazandaran University of Medical Sciences, Sari, Iran; ^a^Both authors contributed equally to the manuscript

**Keywords:** Angiogenic Factors, ELF–PEMFs, hRPE Cells

## Abstract

**Purpose:**

Emerging evidence implies that electromagnetic fields (EMFs) can negatively affect angiogenesis. In this regard, the effects of extremely low frequency pulsed electromagnetic field (ELF–PEMF) exposure on the relative expression level of angiogenic factors involved in the pathogenesis of ocular disorders were evaluated in human retinal pigment epithelial (hRPE) cells in order to investigate a noninvasive therapeutic method for patients with several ocular diseases associated with neovascularization.

**Methods:**

After separating hRPE cells from globes, hRPE cells were exposed to 15 mT of ELF–PEMF (120 Hz) at 5, 10, and 15 min for seven days. Cell proliferation and apoptosis of treated cells were evaluated via ELISA assay. Moreover, relative expression changes of HIF-1α, CTGF, VEGFA, MMP-2, cathepsin D, and E2F3 were performed using real-time RT-PCR.

**Results:**

ELF–PEMF exposure had no significant effects on the apoptosis and proliferation rate of hRPE cells. Expression level of HIF-1α, CTGF, VEGFA, MMP-2, cathepsin D, and E2F3 was downregulated following 5 min of ELF–PEMF exposure.

**Conclusion:**

As ELF–PEMF showed inhibitory effects on the expression of angiogenic genes in hRPE cells with no cytotoxic or proliferative side effects, it can be introduced as a useful procedure for managing angiogenesis induced by retinal pathogenesis, although more studies with adequate follow-up in animal models are needed.

##  INTRODUCTION

Neovascularization, a process of forming new vessels occurs during physiological development and also pathological events, involved in several ocular diseases such as age-related macular degeneration (AMD), ischemic retinal vein occlusion, glaucoma, corneal neovascularization secondary to chemical injury or infection, diabetic retinopathy (DR), and inflammatory processes.^[1,2,3]^ Through the production of different fibrotic and angiogenic factors in choroidal neovascular membranes, retinal pigment epithelial (RPE) cells contribute to choroidal neovascularization (CNV) and paracrine signaling between choriocapillaris and RPE layer.^[4,5,6,7]^ Hypoxia is an important regulator of cell migration and angiogenesis, especially under pathologic conditions, which perform its action by upregulating hypoxia-inducible factor 1 (HIF-1) and vascular endothelial growth factor A (VEGFA).^[8,9,10,11]^ VEGFA mainly regulates angiogenesis, contributing to the migration and proliferation of vascular endothelial cells and tube formation, which increase vascular permeability in angiogenesis.^[12]^ The VEGF/VEGFR signaling pathway is involved in the activation of multiple angiogenic factors, including matrix metalloproteinases (MMPs), cathepsins, connective tissue growth factor (CTGF), and E2Fs.^[13,14]^ CTGF can help regulation of the extracellular matrix (ECM) turnover and a relationship is found between CTGF and CNV.^[15]^ In addition, matrix-degrading proteinases including MMPs and cathepsins induce angiogenesis through facilitating endothelial cell penetration in the subendothelial matrix.^[16,17]^

Suppression of relative angiogenic factors in ocular pathologic conditions can prevent angiogenesis and visual impairment.^[18,20]^ In the treatment of proliferative ocular diseases, anti-VEGF is applied as an intraocular agent.^[21]^ Besides, in many studies, HIF-1^[22]^ and MMPs^[23]^ was introduced as therapeutic targets for retinal-related neovascularization diseases

Several studies have shown that electromagnetic fields can be used as therapeutic options, implicating the biological effects of extremely low frequency pulsed electromagnetic fields (ELF–PEMFs) with respect to their amplitude, frequency, and exposure time.^[24,25,26]^ Many investigations have demonstrated the effects of ELF–PEMFs on the apoptosis, proliferation, differentiation, angiogenic and metastatic properties of different cells.^[27,28,29]^ This study aimed to determine the ELF–PEMF effects on the angiogenic factors in human RPE (hRPE) cells in different exposure times.

##  METHODS 

### Sample Preparation and Cell Culture

Four eye globes from four human neonatal donors with the age of 2–12 months and death enucleation time of < 24 hr, were provided by the Central Eye Bank of Iran. Then posterior segments were cut by removing the iris and vitreous body carefully. Thereafter, the sensory retina was removed after the posterior eyes were dissected into four quadrants. After removing the RPE/choroid layer from the sclera, incubation was carried out for 60 min in 2% dispase solution with 5% CO${}_{2}$ at 37°C. Then, centrifugation was performed at 300 g for five min at 4°C in order to achieve cell pellet. Cell pellet was cultured in 25 cm${}^{2}$ flasks with DMEM/F12 medium (Sigma-Aldrich, Germany), supplemented with 10% FBS at 37°C with 5% CO${}_{2}$. Finally, the confluent cells from passages 2–5 were used for all the experiments.

### Immunocytochemistry

Immunocytochemistry with hRPE cell-specific antibodies was performed in order to characterize hRPE cells from the donated eyes. After culturing the hRPE cells at 5×10${}^{3}$ cells/well in 24-well plates, they were fixed by methanol (–10°C) at room temperature for 10 min. Then, the cells were permeabilized using Triton X-100 (0.25%) and blocked with 1% bovine serum albumin in PBS at room temperature for 60 min.

After irrigating with PBS to remove the blocking agent, the cells were incubated with a 1:1000 mouse monoclonal IgG2a anti-human cytokeratin 8/18 antibody labeling epithelial cells, as well as a 1:100 rabbit polyclonal IgG anti-human RPE65 antibody labeling RPE microsomal membranes (Santa Cruz, CA, USA) overnight at 4°C. The fluorescein isothiocyanate (FITC)-conjugated antibodies (1:100 goat anti-mouse and anti-rabbit IgGs; Santa Cruz, CA, USA) were added for 45 min in darkness at room temperature following irrigation with PBS. Finally, to stain the nucleus, the cells were incubated with 1.5 mg/ml of DAPI (Santa Cruz, CA, USA) for 10 min. To capture images of the labeled cells, an inverted fluorescence microscope (Olympus IX71, Japan) was used with two filters for FITC-conjugated antibodies and DAPI (520 and 460 nm, respectively).

### ELF–PEMF Treatment

Similar to previous studies on PEMFs, an exposure system was provided by Shahid Beheshti University of Medical Sciences (Department of Nuclear Medicine, Tehran, Iran). The magnetic field device contains a controller, and Helmholtz coils consisted of two parallel identical coils (22 cm apart) at 22 cm radius of the curvature. Each coil was constructed by winding 800 turns of 1-mm insulated soft copper wire. The flasks with the confluent hRPE cells was placed between the polar faces of the coils at 37°C with 5% CO${}_{2}$, and exposure time on each day was 5, 10, and 15 min for three cultures. The experiment was performed at a magnetic intensity of 15 mT (120 Hz, 92 V) during one week. Immediately after exposure, ELISA assays and RNA extraction were done.

### ELISA Cell Proliferation and Cell Death Assays

Following ELF–PEMF exposure to the cultured hRPE cells, proliferation and apoptosis of cells were assessed using ELISA BrdU and ELISA PLUS kits (Roche, Germany) based on the instructions, respectively. For this experiment, the cultured hRPE cells in passage two were used at a cell density of 1×10${}^{3}$/ml in a 96-well plate. Proliferation and apoptosis assays of treated cells and control cells were performed in triplicate per cultivated hRPE cell from each donated eye. With respect to cell death ELISA assay, the reading results of the ELF–PEMF-treated cells were compared to the untreated cells and the positive control (a complex of DNA and histone) included in the kit. Absorption of the samples was read at specific wavelengths, using an ELx 808 absorbance reader (BioTek Instruments, VT, USA).

### RNA Isolation and Real-Time RT-PCR

In order to analyze gene expression changes of HIF-1α, CTGF, VEGFA, MMP-2, cathepsin-D, and E2F3 in ELF–PEMF-treated hRPE cells total RNA extraction of the treated and control cells was performed by TRIzol reagent (Ambion, USA). The cells were incubated for five min using TRIzol at room temperature. After adding chloroform to separate RNA, isopropanol (500 μl) was added for RNA precipitation and rinsed with 75% ethanol. Finally, using nuclease-free water, RNA pellet was dissolved. Spectrophotometry analysis was performed using a NanoDrop (Thermo Fisher Scientific, USA) to determine the concentration and purity of the extracted RNAs (A260/280 concentrations). RNA integrity was evaluated by clearly observing 28S and 18S rRNA bands using agarose gel electrophoresis.

The SuperScript reverse transcriptase kit and oligo dT primers (Invitrogen, USA) were used for reverse transcription synthesis of the extracted RNAs in both treated and control cells. Afterward an EvaGreen master mix (Solis BioDyne, Estonia) was used for real-time RT-PCR assay. Specific primers for relative genes and GAPDH, as a housekeeping gene, were used, and efficiency (E) was evaluated for each primer according to the slope of the standard curve. Table 1 presents the primer sequences.

**Table 1:** Primers used in real-time RT-PCR analysis


**Name**	**Forward**	**Reverse**
GAPDH	ACAGTCAGCCGCATCTTC	CTCCGACCTTCACCTTCC
VEGFA	ACTTCTGGGCTGTTCTCG	TCCTCTTCCTTCTCTTCTTCC
CTGF	GCAGGCTAGAGAAGCAGAGC	ATGTCTTCATGCTGGTGCAG
MMP-2	TGGCAAGTACGGCTTCTGTC	TTCTTGTCGCGGTCGTAGTC
E2F3	GAAAGCCCCTCCAGAAACAAG	GCTATGTCCTGAGTTGGTTGAAG
HIF-1α	AACTGGAGACACAATCATATCTTTAG	TTCAGCGGTGGGTAATGGAG
Cathepsin D	TGTGGAGGACCTGATTGC	CGAAGACGACTGTGAAGC

The PCR conditions included an amplification cycle of 94°C for 12 min; 40 cycles of denaturation, amplification, and quantification for 15 s at 95°C, 58-64°C for 30 s, and finally 72°C for 25 s. Also, melting curve analysis was performed at 65°C to 95°C with a gradual increase. RT-PCR assay was carried out in three separate analyses. Every sample was examined in duplicate.

### Statistical Analysis

The results of the ELISA and real-time RT-PCR are presented as mean ± SD of three separate experiments. Kolmogorov Smirnov test was used to check the normal distribution of the data. To compare ELF–PEMF-treated hRPE cultures and control groups, we used Mann–Whitney or *t*-test. *P* < 0.05 was considered statistically significant.

##  RESULTS 

### hRPE Cell Identification

**Figure 1:**
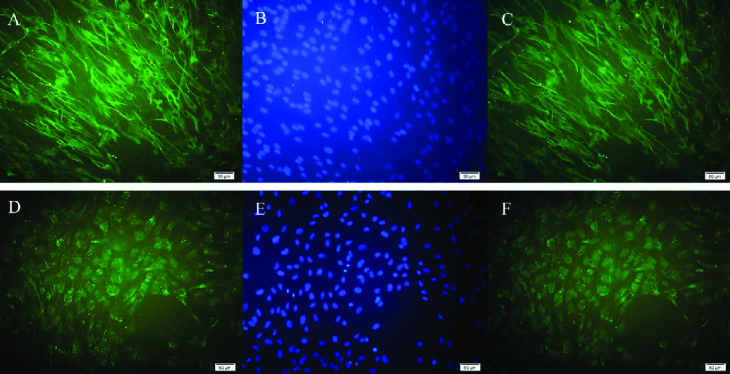
Immunocytochemistry of hRPE cells indicating RPE cell identity. Cytokeratin 8/18 expression confirms the epithelial origin of the cultures, and RPE65 expression confirms that isolated cells are RPE cells. (A) RPE cells stained positively for the fluoresceinisothiocyanate (FITC)-conjugated cytokeratin antibody (green). (B) Nuclei stained blue with 4,6-diamidino-2-phenyindole dihydrochloride (DAPI). (C) Merged image (FITC-labeled cytokeratin and DAPI; 200X). (D) RPE cells stained positively for the RPE65 antibody (green). (E) DAPI-stained RPE cell nuclei (blue). (F) Merged image (FITC-labeled RPE65 and DAPI; 200X).

The RPE cells were cultured in a DMEM/F12 medium (1:1), containing FBS 10% (v/v). After removing the culture medium at 80% confluence within one week, it was subjected to the mentioned ICC protocol. The cultured hRPE cells showed immunoreactivity for cytokeratin 8/18 (Figure 1A-1C) and RPE65 (Figure 1D-1F), confirming their identity.

**Figure 2:**
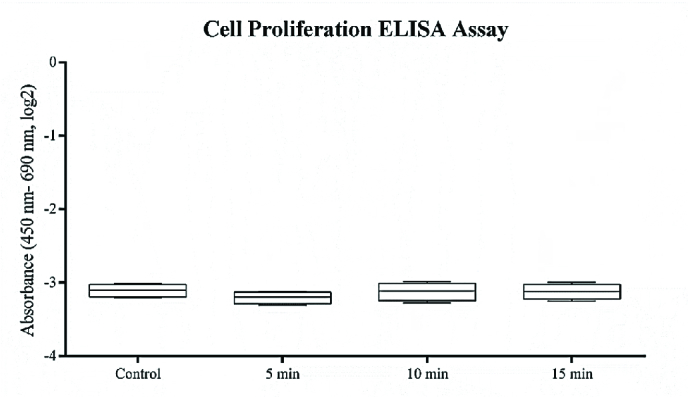
Effect of ELF–PEMF on the proliferation rate of hRPE cells. hRPE cells exposed to ELF–PEMF; After 7 days and each day 5, 10, and 15 min of exposure at a magnetic intensity of 15 mT (120 Hz, 92 V), Cultures were harvested and proliferation assay was performed according to the manufacturer's instructions for cell proliferation ELISA assay. Proliferation rate of hRPE cell cultures did not changed under exposure to ELF–PEMF compared to non-treated control cultures (*P* > 0.05).

**Figure 3:**
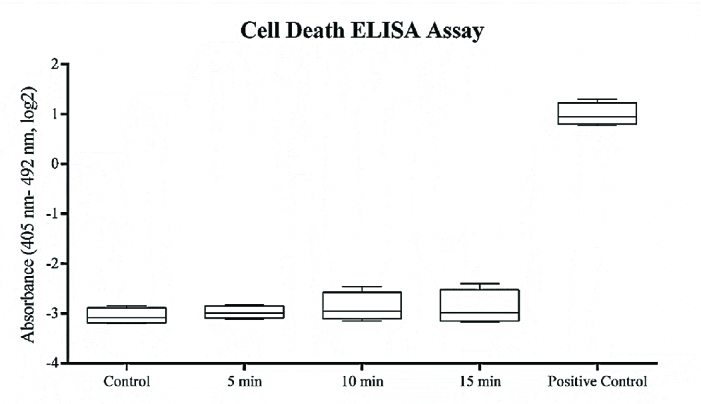
Exploring the cytotoxic effects of ELF–PEMF on hRPE cells. Exposure time on each day was 5, 10, and 15 min and was performed at a magnetic intensity of 15 mT (120 Hz, 92 V) during one week. After exposure, cultures were harvested and subjected to cell death assay according to the manufacturer's instructions for cell death ELISA assay. Results indicated that ELF–PEMF did not has any cytotoxic effects on the treated hRPE cells compared to the positive control provided by the kit (*P* > 0.05).

**Figure 4:**
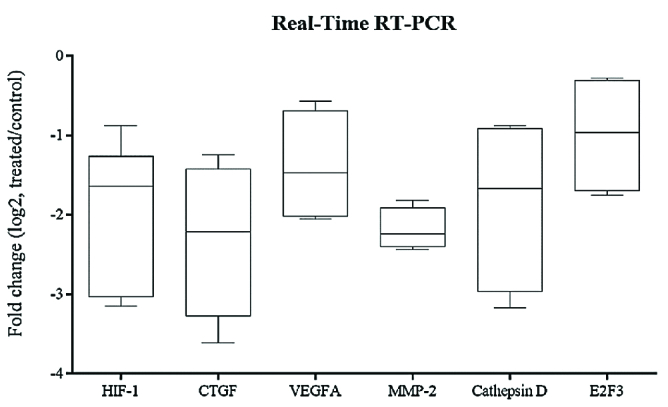
Box plot analysis of HIF-1α, CTGF, VEGFA, MMP-2, cathepsin D and E2F3 expression in hRPE cell cultures exposed to ELF–PEMF as treated cultures compared to hRPE cell cultures without ELF–PEMF as control cultures. After 5 min, RNA was extracted, and gene expression analysis was performed by quantitative real-time RT–PCR as described in the methods section. mRNA levels were normalized to GAPDH and presented as log2 fold change of the control values. Expression levels of all the genes were significantly reduced in the treated hRPE cells compared to the control cultures (*P* < 0.05).

**Figure 5:**
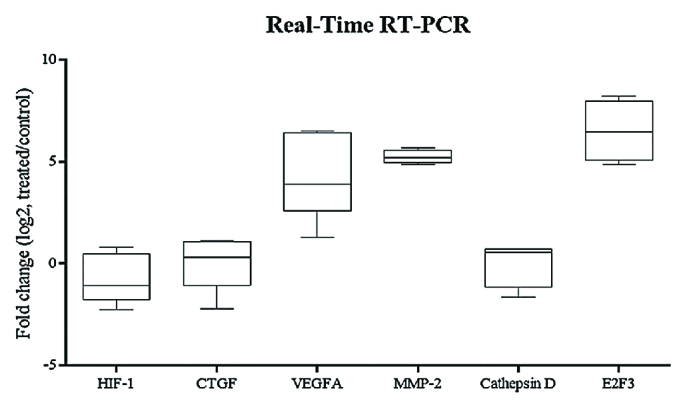
Box plot analysis of HIF-1α, CTGF, VEGFA, MMP-2, cathepsin D and E2F3 expression in hRPE cell cultures exposed to ELF–PEMF as treated cultures compared to hRPE cell cultures without ELF–PEMF as control cultures. After 10 min, RNA was extracted, and gene expression analysis was performed by quantitative real-time RT–PCR as described in the methods section. mRNA levels were normalized to GAPDH and presented as log2 fold change of the control values. Expression levels of VEGFA and MMP-2 were significantly increased in hRPE cells after ELF–PEMF exposure compared to the controls (*P* < 0.05).

**Figure 6:**
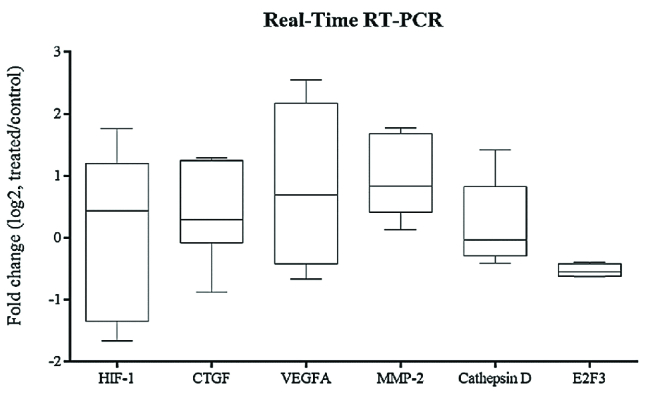
Box plot analysis of HIF-1α, CTGF, VEGFA, MMP-2, cathepsin D and E2F3 expression in hRPE cell cultures exposed to ELF–PEMF as treated cultures compared to hRPE cell cultures without ELF–PEMF as control cultures. After 15 min, RNA was extracted, and gene expression analysis was performed by quantitative real-time RT–PCR as described in the methods section. mRNA levels were normalized to GAPDH and presented as log2 fold change of the control values. Expression levels of CTGF, VEGFA and MMP-2 were significantly increased, while E2F3 was significantly decreased in treated hRPE cells compared to the controls (*P* < 0.05).

### Cell Proliferation and Cell Death Evaluation

Effects of ELF–PEMF on the cell proliferation and apoptosis of hRPE cells were examined by ELISA. The findings showed that ELF–PEMF did not influence cell proliferation (Figure 2). As well, no cytotoxic effect was reported in the treated cells in comparison with the controls (Figure 3).

### Gene Expression Analysis

The expression levels of HIF-1α, CTGF, VEGFA, MMP-2, cathepsin-D, and E2F3 after ELF–PEMF exposure of hRPE cells in comparison with the controls were evaluated by real-time RT-PCR. Effect of ELF–PEMF on mRNA expression was evaluated in three intervals (5, 10, and 15 min). The expression level of all genes significantly reduced in hRPE cells in comparison with the controls after 5 min (Figure 4 and Figure 5, P< 0.05). At 10 min, the expression levels of VEGFA and MMP-2 were significantly increased in hRPE cells after ELF–PEMF exposure compared to the controls. However, after 15 min, only the expression level of E2F3 decreased while CTGF, VEGFA and MMP-2 were significantly increased in treated hRPE cells in comparison with the control group (Figure 6).

##  DISCUSSION

ELF–PEMF exposure had not any cytotoxic and proliferative effects on the hRPE cells, that in combination with negative impacts on the angiogenic factors, the future of this noninvasive treatment strategy for neovascular eye diseases will be promising. The balance between proangiogenic and antiangiogenic factors is essential for normal angiogenesis. However, in pathogenic conditions, the imbalance is perturbed.^[30]^ Pathological retinal angiogenesis induced by hRPE cells in diseases, such as AMD and DR, eventually leads to visual loss. Therefore, it is necessary to be controlled and treated.^[31]^

VEGFA as a key molecule in ocular diseases ^[32]^, is a downstream factor of HIF-1 transcription factor. HIF-1 can increase the expression of VEGFA that promotes cell proliferation and migration through its related receptors.^[33]^ In addition, VEGFA is an upstream factor of CTGF and can upregulate its expression, which is a proangiogenic factor in several organs. Furthermore, it is linked to pathological fibrosis, such as vitreoretinal disorders including AMD and DR.^[15,34,35]^ It was demonstrated that in diabetic rats, the expression level of CTGF was upregulated in the retina.^[36]^ Moreover, the expression of many angiogenic mediators, such as cathepsins and MMPs, is induced by VEGFA and CTGF.^[37,38]^ In addition, in the early phases of angiogenic process, ECM degradation by MMPs and cathepsin proteases is commonly performed.^[39]^

There is an increasing interest in the therapeutic potential of ELF–PEMFs for patients with diseases associated with neovascularization.^[27,40,41]^ It was previously reported that ELF–PEMFs negatively affect angiogenesis in breast cancer.^[42]^ In addition, it was demonstrated that VEGFR2 expression and activation, which is an important factor in vascular formation by endothelial cells, decreased following ELF–PEMFs treatment.^[26]^

Moreover, it was shown that ELF–PEMFs activate stress proteins, including heat-shock proteins (e.g., HSP90, and HSP70), changing the half-life, activity or expression of proteins, such as VEGFR1 and VEGFR2.^[27,43,44,45,46,47]^ In addition, it was demonstrated that ELF–PEMF stimulation significantly decreased the renal expression of VEGFA.^[48]^ In the current study, ELF–PEMF negatively affected the expression of VEGFA, HIF-1α, CTGF, MMP-2, cathepsin-D, and E2F3 as angiogenic factors. Therefore, ELF–PEMF can be investigated as a novel therapeutic approach for retinal neovascular disorders. The mentioned antiangiogenic effect was after 5-min treatment, at 10 min, results were reversed and ELF–PEMF acted as an angiogenic agent! VEGFA and MMP-2 were increased significantly in the treated cultures. At 15 min, in addition to VEGFA and MMP-2, CTGF significantly was increased compared to the untreated cultures. Based on the results, the effect of ELF–PEMF on hRPE cells is time-dependent. Besides, in different articles effects of ELF–PEMF was shown to be dependent on more parameters such as cell type, frequency and flux density.${}^{\left[48,49\right]}$ Emerging evidence suggests the use of ELF–PEMF in therapy still requires a lot of research.

##  CONCLUSION

We observed that ELF–PEMFs have significant inhibitory roles on the expression of angiogenic factors in the hRPE cells, as pivotal cells in ocular health and disease. Therefore, ELF–PEMFs can be used for therapeutic applications in ocular diseases, but field strengths, frequencies, and exposure times must be considered. Also, further studies should be done to evaluate important genes and factors altered by ELF–EMF in different settings and situations.

##  Financial Support and Sponsorship

The current study has been derived from a research project funded by Stem Cell Research Center, Golestan University of Medical Sciences, Gorgan, Iran (Grant number: 950804171).

##  Conflicts of Interest

All authors declare that they have no conflicts of interests.
